# The genus *Neotherina* Dognin (Geometridae, Ennominae) in Costa Rica

**DOI:** 10.3897/zookeys.149.2346

**Published:** 2011-11-24

**Authors:** J. Bolling Sullivan, Isidro Chacón

**Affiliations:** 1 200 Craven St., Beaufort, North Carolina 28516 USA; 2 Instituto Nacional de Biodiversidad, Heredia, Santo Domingo, Costa Rica

**Keywords:** Taxonomy, Ourapterygini, Nephodiini, *Neotherina*, *Nephodia*, Costa Rica

## Abstract

So far, two species of *Neotherina* Dognin have been recorded in Costa Rica. *Neotherina imperilla* (Dognin) occurs primarily at altitudes between 1100 and 1700 meters and *Neotherina callas* (Druce) which is widely distributed above 1100 meters. A third, new species, *Neotherina xanthosa* Sullivan and Chacón is described from altitudes above 2400 meters. Heterogeneity of the genus is discussed.

## Introduction

The neotropical ennomine genus, *Neotherina* Dognin, contains eight species, four of which were recently moved into the genus from other genera (Pitkin 2000). One additional species appears to be misplaced in the genus but so far, no apomorphic characters have been defined for *Neotherina*. Superficially, the species look like species currently placed in *Nephodia* Hübner and *Lambdina* Capps, both of which likely are paraphyletic assemblages. Pitkin (2002) figures the adult male and female genitalia of *Neotherina callas* (Druce), and the male genitalia of *Neotherina imperilla* (Dognin) from the Central Cordillera of Colombia. The latter is currently considered to be the senior subjective synonym of the type species, *Neotherina inconspicua* Dognin, described from Lino, Panama. Pitkin et al. (1996) list the species of *Neotherina* occurring in Costa Rica (*Neotherina callas*, *Neotherina imperilla*) and state that there is a third, possibly undescribed, species there too. It is this latter species that we describe here, and also discuss the generic relationships of the species currently placed in *Neotherina*.

## Materials and methods

Photographic methods used herein are described in Sullivan and Adams (2009). Procedures for dissecting and preparing genitalia follow those of Lafontaine (2004). DNA sequencing of the barcode fragment of the COI gene was carried out at the Canadian Center for DNA Barcoding, Guelph, Ontario. Barcode sequences were compared by nearest neighbor analyses as implemented on the Barcode of Life Data systems website (Ratnasingham and Hebert 2007).

### Repository abbreviations

Specimens were examined from the following collections:

INBio Instituto Nacional de Biodiversidad, Santo Domingo de Heredia, Costa Rica

JBS J. Bolling Sullivan, Beaufort, North Carolina, USA

USNM National Museum of Natural History, Washington, District of Columbia, USA

## Systematics

### Neotherina

Dognin

http://species-id.net/wiki/Neotherina

Neotherina Dognin, 1914: 402. Type species, *Neotherina inconspicua*, Dognin, 1914.

#### Remarks. 

Pitkin (2002) indicated that *Neotherina* has no apomorphies that adequately define it and that it is closely related to *Evita* Capps, *Lambdina* and *Nepytia* Hulst. She transferred four species into the genus from other genera (*Trygodes* Guènee, *Eusarca* Hübner) based in large part on the structure of the aedeagus (pointed, sinuous, posterior process and usually with a subterminal process as well). She considered the transfer provisional based on the uncertainly of the monophyly of *Neotherina*. One species, *Neotherina noxiosa* Dognin, was removed from the genus by Pitkin because it lacks a furca, a process originating near the dorsal margin of the juxta that defines the Ourapterygini into which *Neotherina* has been placed. Superficially, *Neotherina* species are similar to species placed in *Lambdina* and *Nephodia* (Nephodiini), but the monophyly of those genera is uncertain. The monophyly of the Ourapterygini versus Nephodiini is also questionable, since characters separating the two tribes are based largely on characters of the furca (Pitkin 2002, see also Sihvonen et al. 2011).

### Neotherina imperilla

(Dognin, 1911)

http://species-id.net/wiki/Neotherina_imperilla

Figure 1

#### Remarks. 

**Figure 1.**
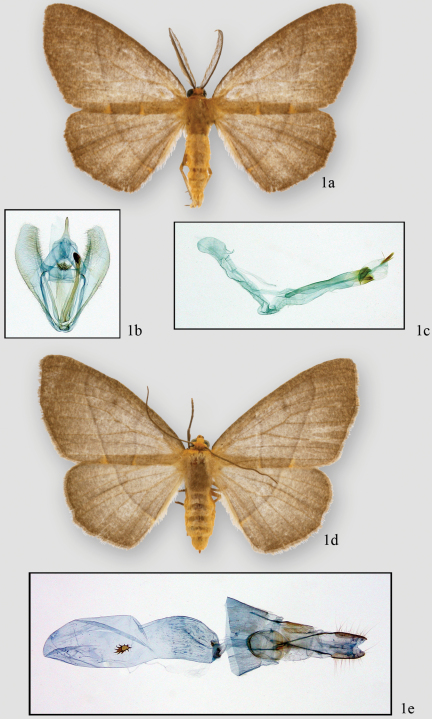
*Neotherina imperilla* male adult **1a** genital capsule **1b** aedeagus **1c** female adult **1d** and female genitalia **1e**

Two specimens in the INBio collections were identified as *Neotherina imperilla* by Linda Pitkin during her work on the Ennominae of Costa Rica (Pitkin et al. 1996). Superficially they resemble the type (USNM) except that the type is quite faded. The species looks very much like those currently placed in the genus *Lambdina*. The ground color is orange brown with distinct medial and postmedial lines crossing the forewings. The scaling on the head, thorax and abdomen is orange with the region between the antennal bases and collar being brighter in color. There is no dorsal tuft on the metathorax. Notable characters include the male bipectinate antennae and small orange spots distal of the junction of the postmedial line and the anal edge of the forewing and proximal to the medial line and anal edge of both wings. There is a very small extension at vein M3 of both wings. The genitalia of a male from the INBio collection (Fig. 1b, c) closely resembles that of the male figured in Pitkin (2002) but there appear to be slight differences in the shape of the uncus and perhaps in the structures of the vesica (not everted in Pitkin (2002). The female genitalia (Fig. 1e) are figured for the first time but there is no female from the type locality at the USNM for comparison. Since few geometrid species are shared between the Costa Rican and South American fauna (Janzen, Brehm and Sullivan, unpubl. data), the taxonomic status of Costa Rican *Neotherina imperilla* should be re-evaluated when more study material becomes available.

#### Diagnosis.

The wing pattern of *Neotherina imperilla* is similar to many species in *Lambdina*, *Nephodia*, and unplaced species that occur at similar altitudes. It may be distinguished by the rounded apex and orange-brown color of the forewings. Similar (probably undescribed) species have a more pointed apex and the ground color is reddish or purplish.

#### Distribution and biology.

Nothing is known about the life history of this species. It has been collected on the western slope of the Cordillera Volcánica de Guanacaste, the western slope of the Cordillera de Tilaran, both western and eastern slopes of the Cordillera Volcánica Central and both slopes of the Cordillera de Talamanca and the Fila Costeña. Most specimens at INBio (44) come from 1100–1700 m on the western slopes but this may reflect the absence of collecting access to eastern slopes above 900 m.

### Neotherina callas

(Druce, 1892)

http://species-id.net/wiki/Neotherina_callas

**Figure 2.**
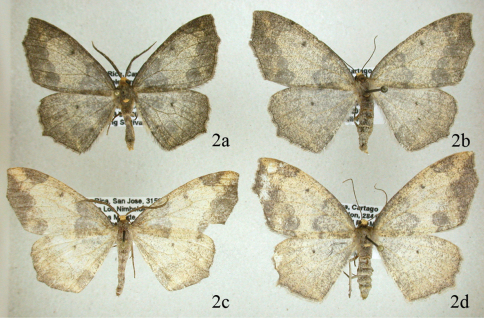
Comparison of *Neotherina callas* male **2a** and female **2b** and *Neotherina xanthosa* male **2c** and female (**2d**) adults.

Figures 2a, 2b 3 5a, 5b, 5d

#### Remarks.

**Figure 3.**
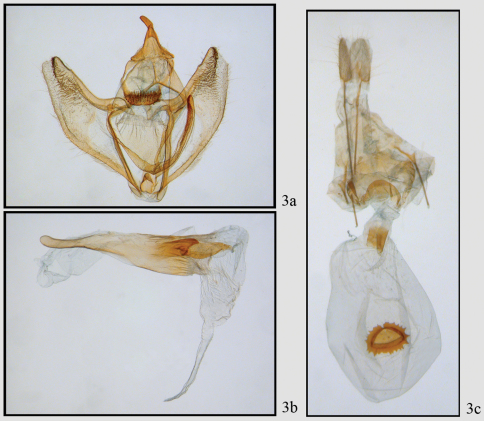
*Neotherina callas* male **3a,b** and female **3c** genitalia.

This moderately common species is found at altitudes between 1100 and 2800 meters throughout Costa Rica. The forewing appears to be truncated at the tip because there are well-developed extensions of vein M3 in both wings; Females are noticeably larger than males. Adults of this and the following species are shown in Fig. 2. The female genitalia were figured by Pitkin (2002) and are shown in Figs 3c, 5a, 5b. There is a well-defined collar on the ductus and a distinctive signum on the bursa. The male genitalia (Figs 3a, 3b, 5d) are typical for the tribe Ourapterygini in having a well-developed furca, but have few other distinguishing characters for tribal classification.

#### Diagnosis.

This species is unlikely to be confused with any other species in Costa Rica except *Neotherina xanthosa* because of the characteristic wing shape. The wings are diaphanous and overlaid by a distinct pattern seen only in this species and in *Neotherina xanthosa* (Fig. 2). It can be separated from the latter by the darker more grayish color and its smaller size, with a male forewing length of 18.95 mm (18–22 mm; *n* = 64) compared to 22.03 mm in *Neotherina xanthosa*; females average 21.64 mm (range 19-24 mm; *n* = 64) versus 23.15 mm in *Neotherina xanthosa*. Genitalic differences are given under the *Neotherina xanthosa* diagnosis.

#### Distribution and biology.

Nothing is known about the life history of this species. There are over 100 specimens in collections (INBio, USNM, JBS) and it occurs throughout Costa Rica at altitudes between 1100 m and 2800 m along all slopes. At higher altitudes the specimens are larger (see Sullivan and Miller 2008).

### Neotherina xanthosa

Sullivan & Chacón sp. n.

urn:lsid:zoobank.org:act:957EF99D-BEF8-4D40-B8AC-2A6458E88450

http://species-id.net/wiki/Neotherina_xanthosa

Figures 2c, 2d 4 5c, 5e

#### Type material.

Holotype male: Costa Rica, Rio Macho. Est. Ojo de Agua. Send. A Torre 47, Cartago Province 2960 m, 26 March 1998, leg. E. Alfaro, B. Gamboa (INBIOCR1002526641) (INBI). **Paratypes:** (male) same data as type (INBIOCR1002603341); (male) same data as type but 24–28 Feb. 1998 (INBIOCR1002526656); (2 females) Costa Rica, Macizo de la Muerte, Sector de la Esperanza, Cartago Province, 2650 m, Nov. 2002, leg. R Delgado (INB0003534645, 0003554631); (male) same as previous but Sept. 2002, INB0003536193); (male) Costa Rica, San Gerardo de Dota, San Jose Province, 2430 m, 23 Dec. 1981, leg. DH and WH Janzen (INB0004269188); (male) Costa Rica, 4.6 Km E. de Villa Mills, Cartago Province, 2600 m, 21–26 Sept. 1995 (INBIOCR1002435795); (2 females) Costa Rica, Estac. Barva, Braulio Carillo N. P., Heredia Province, 2500 m, G. Rivera (INBIOCR1000089203, 1000089215); (2 females) same, Jan. 1990 (INBIOCR1000121385; 1000206721); (female) same, Feb. 1990 (INBIOCR1000157034); (female) same but leg. A. Fernandez, Nov. 1989 (INBIOCR1000156409); (female) same, Feb. 1990 (INBIOCR1000125703); (female) same, Apr. 1990 (INBIOCR1000169281) (5 females) same but leg. B. Apu & G. Varela, June 1990 (INBIOCR100220347, 100225846, 1000225866); (male, female) Costa Rica, Est. Los Nimbolos, Cerro de la Muerte, San Jose Province, 3150 m, 24–27 Jul. 2008, J.B. Sullivan, (female) Costa Rica, Villa Mills, Cartago Province, 2841 m 19–21 Mar 2010, J.B. Sullivan (GenBank accession number JF855656)(INBio, JBS, USNM).

**Figure 4.**
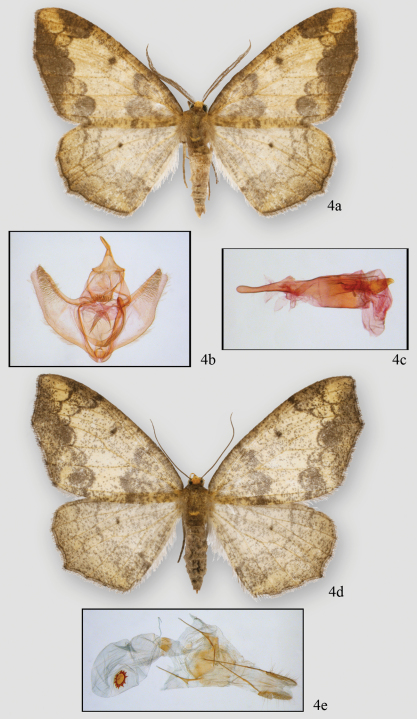
*Neotherina xanthosa*, male holotype **4a** and male genitalia (**4b.c** female paratype 4d and female genitalia 4e.

**Figure 5.**
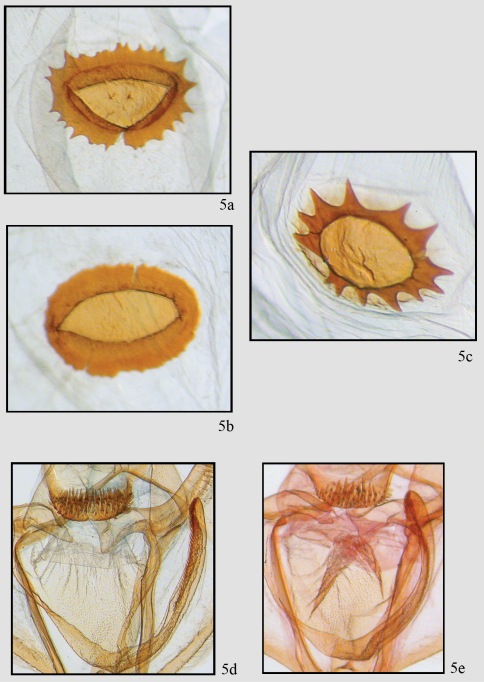
Comparison of genitalic structure in *Neotherina callas* and *Neotherina xanthosa*. Two extremes of female signum **5a, b** of *Neotherina callas* compared to *Neotherina xanthosa* **5c** detail of male gnathos and furca of *Neotherina callas* **5d** and *Neotherina xanthosa* **5e.**

#### Etymology.

The name refers to the yellowish-brown ground color of the maculation.

#### Diagnosis.

The species is similar only to *Neotherina callas*, which it can be distinguished from by its yellowish-brown color and larger size (Fig. 2). Certain identification is best made by dissection of a male and examination of the spinulose terminal portion of the furca. In *Neotherina xanthosa* it is about half the length of the furca (Fig. 5e), whereas in *Neotherina callas* it is approximately one fourth as long as the furca (Fig. 5d). The female signa on the bursae differ in shape as well (compare Figs 5a, 5b, 5c). *Neotherina xanthosa* also differs from *Neotherina callas* (GenBank accession numbers JF855657; JF855658; JN268704; HM878904) by 5.6% in its DNA barcode.

#### Description.

**Male.** Fig. 2c, 4a,b,c, 5e. *Head* – Palps very small, barely extending above middle of eye, scaling straw colored basally becoming chocolate on 2^nd^ and 3^rd^ segments. First segment more than 2 X length of second segment which is more than 2 X length of third segment. Frons brown yellow, square, yellow extending to collar; eyes hemispherical; ocellus absent; tongue normal. Antennae bipectinate, pectinations long at base (5 × shaft width) tapering distally to unpectinated discs in last 8 segments (56–58 segments); bipectinations toward apex with rami swollen distally, more basal bipectinations tubular, not swollen distally. Rami almost chocolate brown, dorsal shaft with scaling brown. Rami originate ventrally just lateral of midline. Scape brown yellow. *Thorax and abdomen*–Scaling slender, brown and off-white, distinct pad of yellow-brown scales at distal end of metathorax. Dorsal abdominal scaling off white and brown, shorter, thicker scales with multiple points distally (usually 3). Underside similar. Terminal scales on each segment brownish forming poorly-differentiated rings. Legs covered with tightly adhering band and brown scales, those of spurs darker, spurs short, epiphysis slender, long but slightly shorter in length than femur and extending slightly past distal end of femur. Leg scaling extremely difficult to remove. Proportions of leg segments typical. *Wings*–Forewing venation with two areoles beyond cell, WL 22.03 mm (21–22 mm, N=7). Wing pattern very similar to that of *Neotherina callas* but ground color in *Neotherina callas* gray, whereas in *Neotherina xanthosa* it is brownish yellow. Forewing tip appears scalloped because M3 is extended and there is a similar but smaller extension at M3 on hindwing. Wings of *Neotherina callas* similar. *Male genitalia* (Fig. 4b,c, 5e)–Uncus slightly hooked, pencil-like, tapering to a broad base and forming an inverted T. Tegumen very broad, vinculum narrow. Gnathos with arms poorly defined but expanding medially to a broad medial area supporting three or more rows of well-defined spines, extending in height to width of medial pad. Small spines along lateral edge of pad. Furca deflects to right bearing hair-like bristles on inner 20–30%. Furca curves medially, rounded tip. Juxta small, basal area with posterior point. Area medial to furca arm granulated. Valva bulging medially, tapering to tip. Costa sclerotized, broad forming blade-like process at tip of valva. Medial 40% of valve with moderately long setae. Anal edge of valve with bulge medially then tapering to subapical tip. Anellar extensions of costa do not join medially. **Female.** Figs 2d, 4d,e, 5c. Antenna filiform, otherwise similar to male but slightly larger (WL 23.15 mm; 22–25 mm; n = 22). *Female genitalia* (Fig. 4e, 5c) – Anal papillae slightly pointed and rounded terminally. Posterior apophyses long, 2 × longer than anterior apophyses. Posterior vaginal plate sclerotized and broadly rounded posteriorly. Anterior plate unsclerotized at base. Ductus bursae short with sclerotized plate dorsally forming collar-like structure. Ductus moderately short. Corpus bursae sac-like with well-defined signum. Dorsal signum round, hollow with star-like basal collar of 13 prongs or points. Center deeply invaginated. Ductus ejaculatorius originates on upper part of corpus bursae below collar on ductus bursae.

#### Distribution.

Known from above 2400 m in the Talamanca and the Central Volcanic ranges in Costa Rica. In flight throughout the year.

#### Remarks.

Nothing is known about the biology of this species, or that of any other *Neotherina* species. Its range probably extends into the other mountain ranges in Costa Rica and northern Panama.

## Discussion

The three species of *Neotherina* now known from Costa Rica form a heterogeneous assemblage. Wing shapes for *Neotherina callas* and *Neotherina xanthosa* are identical, but very different from those of *Neotherina imperilla*. Of the remaining species, *Neotherina melia* (Druce), *Neotherina simplissima* (Dyar) and *Neotherina atomeria* (Schaus), currently a synonym of *Neotherina callas*, are extremely similar to *Neotherina callas* and may be conspecific. *Neotherina axona* (Druce), *Neotherina consequens* (Prout), *Neotherina inconspicua* (Dognin) (currently a synonym of *Neotherina imperilla*), *Neotherina nomia* (Druce) and *Neotherina carbania* (Druce) seem to be a heterogeneous assemblage but we have not dissected nor barcoded most of them. The genitalia examined to date do not present characters apomorphic for *Neotherina*. Barcoding of geometrid specimens from Costa Rica and Ecuador (Janzen, Sullivan, Brehm, unpubl. data) has revealed very few shared species. Likewise, genital dissections show little overlap between apparent conspecific specimens from western Colombia and Costa Rica (Sullivan, unpubl. data). Additional collections are needed to determine if populations of supposed *Neotherina imperilla* from Costa Rica are conspecific with those from the type locality, Mt. Tolima, in Colombia.

*Neotherina callas* and *Neotherina xanthosa* together with *Neotherina melia*, *Neotherina atomaria*, and *Neotherina simplissima* seem to form a natural group. When additional data on food plants, barcodes, and genitalia of the remaining species currently placed in *Neotherina* are available, the “*callas* complex” may require a new genus.

## Supplementary Material

XML Treatment for Neotherina

XML Treatment for Neotherina imperilla

XML Treatment for Neotherina callas

XML Treatment for Neotherina xanthosa
